# Early vs. delayed neurological improvements in acute ischemic stroke patients following endovascular treatment

**DOI:** 10.1515/tnsci-2025-0395

**Published:** 2026-10-22

**Authors:** Zunbao Xu, Mohammad Mofatteh, Zijie Zheng, Zhenzhen Zhu, Biqing Yang, Wanyi He, Rixin Luo, Robert W. Regenhardt, Zhenzhang Li, Qiaowei Li

**Affiliations:** Neuromedical Center Ward 2, The Affiliated Panyu Central Hospital, Guangzhou Medical University, Guangzhou, China; Geriatric Medicine Institute of Panyu District, Guangzhou, China; School of Medicine, Dentistry and Biomedical Sciences, Queen’s University Belfast, Belfast, UK; School of Automation Science and Engineering, South China University of Technology, Guangzhou, China; Department of Neurology, Neurosurgery, Massachusetts General Hospital, Harvard Medical School, Boston, MA, USA; College of Mathematics and Systems Science, Guangdong Polytechnic Normal University, Guangzhou, China; School of Basic Medical Sciences, Guangzhou Medical University, Guangzhou, China

**Keywords:** endovascular treatment, acute ischemic stroke, early neurological improvement, patient outcome, NIHSS

## Abstract

**Objectives:**

Early neurological improvement (ENI) at 24 h after endovascular treatment (EVT) is a surrogate marker for predicting functional improvement of acute ischemic stroke (AIS) patients; however, using delayed neurological improvement (DNI) at seven days post-EVT remains understudied. We investigated differences between ENI and DNI in predicting functional outcome of AIS patients undergoing EVT.

**Methods:**

We retrospectively analyzed the prospectively collected data from 187 patients undergoing EVT. ENI was defined as a reduction of more than four points on the National Institute of Health Stroke Scale (NIHSS) after 24 h compared with admission or an NIHSS of 0 or 1 after 24 h; whereas the same parameter at seven days post-EVT was defined as DNI. A favorable outcome was a modified Rankin scale (mRS) score of 0–2 after 90 days.

**Results:**

There were no statistically significant differences in sex, age, hypertension, diabetes, coronary artery disease, atrial fibrillation, hyperlipidemia, successful recanalization (modified thrombolysis in cerebral infarction (mTIC)=2b-3), complete recanalization (mTICI=3), pre-EVT NIHSS, bridging thrombolysis, door-to-puncture time, last-known well time, and parenchymal hematoma. 62.96 % of patients in the DNI group achieved favorable outcome compared to 78.21 % in the ENI group (p=0.118).

**Conclusions:**

DNI may be used similarly to ENI to predict favorable outcomes post-EVT.

## Introduction

Stroke is the second leading cause of mortality worldwide and a leading cause of disability, with increasing incidence in developing countries [1]. According to the report published by the World Stroke Organization, more than 13.7 million strokes are reported annually, with an annual mortality of 2.7 million [2], 3].

The majority of strokes, accounting for 85 % of cases, are caused by arterial occlusion. Therefore, the management focuses on arterial reperfusion to reduce mortality and morbidity [2], 3]. Approximately 11–29 % of acute ischemic stroke (AIS) cases are caused by large vessel occlusions (LVO), involving the internal carotid artery (ICA), M1 or M2 segment of the middle cerebral artery (MCA), or vertebrobasilar system [1], 4], 5]. Brain hypoperfusion results in penumbra, or region at risk tissue, and core infarction, or region of irreversible tissue death [6], 7].

Treatment of AIS patients has improved significantly over the past few decades, and endovascular treatment (EVT) has become gold-standard in many healthcare centers globally to achieve recanalization of LVO [8], 9]. Identifying markers of neurological improvement in patients with LVO undergoing EVT can be crucial in predicting longer-term outcomes. Multiple previous studies reported that prediction of functional outcome of AIS patients can be achieved by early neurologic improvement (ENI) [10], [11], [12], [13], [14], [15]. It has been used as a surrogate for long-term outcome following EVT, defined as National Institute of Health Stroke Scale (NIHSS) score improvement of greater than four points or NIHSS of zero or one at 24 h post-EVT [16]. ENI is associated with restoration of the blood circulation and flow improvement in collateral vessels [17], 18]. However, a major caveat is that using such early surrogates within 24 h cannot adequately reflect patients’ stable condition in the long-term. For example, common complications can arise after 24 h following EVT, resulting in patient’s deterioration and failure of accurate outcome prediction [19]. Therefore, several studies suggested that in contrast to ENI (NIHSS score at 24 h post-EVT), NIHSS on the seventh day post-EVT, called delayed neurological improvement (DNI) could be a better parameter for predicting clinical outcomes [19], 20].

As there is a lack of consensus on the usage of ENI vs. DNI, the aim of the current study was to identify potential differences between ENI and DNI for AIS patients following EVT.

## Materials and methods

### Patient population

We conducted a retrospective analysis of prospectively collected data from consecutive patients who underwent EVT in a comprehensive stroke center in China between 2019 and 2022.

Inclusion criteria were the following: 1) patients who underwent EVT; 2) age ≥18 years old at the time of treatment; 3) within 24 h from last know well time (LKW); 4) premorbid modified Rankin scale (mRS) of lower than 2. Exclusion criteria were as follows: 1) missing follow-up data; 2) pre-EVT NIHSS less than 6; 3) pre-EVT Alberta stroke program early CT score (ASPECTS) less than 6; 4) lack of early or delayed neurological improvements.

### Study design and data collection

#### Outcome measures

In our study, ENI was defined as a reduction of more than four points on the NIHSS score after 24 h compared with admission or an NIHSS of zero or one after 24 h [21]. In contrast, DNI was defined as a reduction of more than four points on the NIHSS score after seven days compared with admission or an NIHSS of zero or one seven days since admission.

The following baseline data were collected in our study: patient characteristics, including sex, age, comorbidities and risk factors (history of prior stroke, hypertension, diabetes, coronary artery disease, atrial fibrillation, hyperlipidemia, successful recanalization (modified thrombolysis in cerebral infarction (mTICI) of 2b-3)) [22] complete recanalization (mTICI of 3), pre-EVT NIHSS, pre-EVT ASPECTS, bridging thrombolysis, door-to-puncture time (DPT), last know well-to-puncture time (LKWPT), and parenchymal hematoma. Also, 3-month outcome was evaluated by mRS, and favorable outcome was considered as mRS scores of 0–2 [23], [24], [25].

#### Ethics approval and consent to participate

The study was approved by The Affiliated Panyu Central Hospital, Guangzhou Medical University Review Board. Informed consent was not required due to the retrospective nature of the study in compliance with The Affiliated Panyu Central Hospital, Guangzhou Medical University Review Board. All procedures performed in the studies involving human participants were in accordance with the ethical standards of the institutional and/or national research committee and with the 1964 Declaration of Helsinki and its later amendments or comparable ethical standards.

#### Statistical analysis

Using IBM SPSS version 26 (IBM-Armonk, NY), the non-parametric Mann-Whitney U test was performed to analyze non-normally distributed continuous data, reported as medians along with the interquartile range (IQR). Categorical variables were described as frequencies and percentages. Results were considered statistically significant if the p value was below 0.05.

## Results

### Patient selection

Initially, 187 consecutive patients were screened for our study. In total, 68 patients were excluded due to the following reasons: missing follow-up (n=5), pre-EVT NIHSS less than 6 (n=12), lack of data to assess ENI or DNI (n=63), and pre-EVT ASPECTS less than 6 (n=2). Therefore, 105 patients were included in final analysis, which were grouped into ENI (n=78, 74.3 %), and DNI (n=27, 25.7 %).

### Study characteristics

Baseline patient characteristics are demonstrated between DNI and ENI in Table 1. There were no statistical differences in sex (p=0.653), age (p=0.294), hypertension (p=0.847), diabetes (p=0.461), coronary artery disease (p=0.674), atrial fibrillation (p=0.467), hyperlipidemia (p=0.720), successful recanalization (mTIC≥2b) (p=0.257), complete recanalization (mTICI=3) (p=0.963), pre-EVT NIHSS (p=0.597), pre-EVT ASPECTS (p=0.210), bridging thrombolysis (p=0.858), DPT (p=0.736), LKWT (p=0.615), parenchymal hematoma (PH) (p=0.092).

**Table 1:** Comparison of baseline characteristics.

	DNI (n=27)	ENI (n=78)	p-Value
Male sex (n, %)	16 (59.26 %)	50 (64.10 %)	0.653
Age year (IQR)	64.00 (58.0, 77.0)	65.00 (55.8, 73.0)	0.294
Hypertension (n, %)	15 (55.56 %)	45 (57.69 %)	0.847
Diabetes (n, %)	7 (25.93 %)	15 (19.23 %)	0.461
Coronary artery disease (n, %)	1 (3.70 %)	6 (7.69 %)	0.674
Atrial fibrillation (n, %)	12 (44.44 %)	41 (52.56 %)	0.467
Hyperlipidemia (n, %)	9 (33.33 %)	29 (37.18 %)	0.720
Successful recanalization (mTICI≥2b) (n, %)	26 (96.30 %)	78 (100.00 %)	0.257
Complete recanalization (mTICI=3) (n, %)	21 (77.78 %)	61 (78.21 %)	0.963
Pre-EVT NIHSS (IQR)	14.0 (7.0, 20.0)	13.0 (10.0, 18.0 %)	0.597
Pre-EVT ASPECTS	9.0 (8.0, 10.0)	10.0 (9.0, 10.0)	0.210
Bridging thrombolysis (n, %)	13 (48.15 %)	36 (46.15 %)	0.858
DPT (IQR), min	89.0 (76.0, 111.0)	96.0 (75.0, 112.0)	0.736
LKWPT (IQR), min	269.0 (177.0, 462.0)	229.0 (162.3, 421.8)	0.615
PH (n, %)	6 (22.22 %)	7 (8.97 %)	0.092

ASPECTS, Alberta stroke program early CT score; DNI, delayed neurological improvement; DPT, door-to-puncture time; ENI, early neurological improvement; EVT, endovascular treatment; IQR, interquartile range; LKWT, last known well-to-puncture time; mTICI, modified thrombolysis in cerebral infarction; PH, parenchymal hematoma.

### Favorable outcome

Our analysis revealed that 17 patients (62.96 %) in the DNI group achieved 90-day favorable outcomes, whereas 61 patients (78.21 %) had favorable outcomes in the ENI group. This difference was not statistically significant (p=0.118). In addition, there were no statistically significant differences between the DNI and ENI groups for 3-months mRS (p=0.140) (Table 2).

**Table 2:** Comparison of 3-month outcome of DNI and ENI.

		DNI	ENI	p-Value
Favorable outcome (n, %)		17 (62.96 %)	61 (78.21 %)	0.118
3-month mRS	0.0	9 (33.33 %)	36 (46.15 %)	0.140
	1.0	6 (22.22 %)	22 (28.21 %)	
	2.0	2 (7.41 %)	3 (3.85 %)	
	3.0	3 (11.11 %)	8 (10.26 %)	
	4.0	7 (25.93 %)	5 (6.41 %)	
	5.0	0 (0.00 %)	1 (1.28 %)	
	6.0	0 (0.00 %)	3 (3.85 %)	

DNI, delayed neurological improvement; ENI, early neurological improvement; mRS, modified Rankin scale.

The distribution of 3-months mRS is shown in Figure 1.

**Figure 1:**
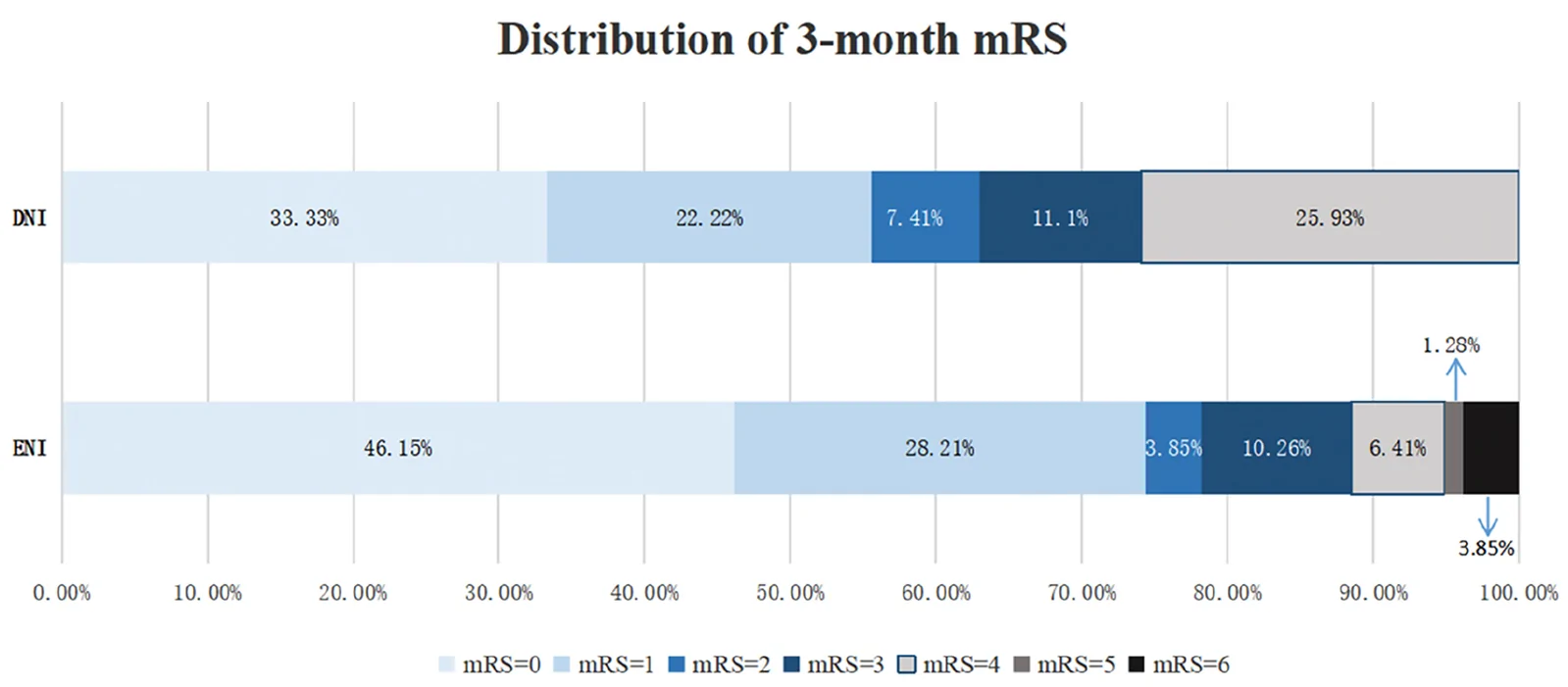
Distribution of 3-months modified Rankin scale (mRS).

## Discussion

The current study aimed to investigate whether there are differences between ENI and DNI in predicting functional outcome of AIS patients undergoing EVT. Our findings revealed that there were no statistically significant differences in favorable outcome between ENI and DNI following EVT. These results may have relevant clinical implications because the probability of delayed neurological recovery in the absence of significant neurological improvements within 24 h post-EVT should not be neglected, and proactive patient care should be maintained regardless of their early neurological course [26], [27], [28], [29]. For example, other studies showed that multiple factors, including a history of diabetes mellitus history, pre-stroke mRS, longer last known well-to-puncture time, lack of mTCI=3, and the number of mechanical thrombectomy attempts were predictors of failure for neurological improvements [21]. Therefore, DNI may be used alternatively to predict functional outcomes in the absence of neurological improvements within 24 h.

DNI is assessed later compared to ENI, and as such may be less influenced by complications after EVT. Therefore, it may reflect patient outcomes more effectively and reliably [20]. Various complications, such as malignant cerebral edema and stroke-associated pneumonia can occur within seven days post-EVT, which can result in an unfavorable prognosis [30], 31], emphasizing the importance of considering neurological improvements beyond 24 h in predicting long term outcomes after EVT. Therefore, both NIHSS improvements at 24 h and 7 days post-EVT may be used reliably by physicians and healthcare providers for clinical decision-making and post-operative care to predict the patient outcomes.

While our study provides valuable insights into outcomes after EVT in AIS, it is important to note its limitations. As a retrospective analysis, the collected data are inherently subject to potential biases commonly associated with retrospective data collection methods. In addition, the study was conducted at a single comprehensive stroke center in China, which can limit the generalizability of our findings to other populations or healthcare settings globally. Despite these limitations, the findings of our study can pave the way for future investigations in the management of AIS patients and prediction of outcomes after EVT. Future large-scale multi-center investigations could focus on prospective studies to validate and extend our findings across diverse demographics and clinical settings. These studies could shed light into understanding the interplay between various factors influencing ENI and DNI.

## Conclusions

In our real-world analysis of patients who underwent EVT, there were no statistically significant differences between ENI at 24 h and DNI at 7 days for predicting 90-day functional outcome of AIS patients undergoing EVT. Therefore, DNI may be used similarly to ENI to predict favorable outcomes post-EVT.
